# 18F-FLT Positron Emission Tomography/Computed Tomography Imaging in Pancreatic Cancer: Determination of Tumor Proliferative Activity and Comparison with Glycolytic Activity as Measured by 18F-FDG Positron Emission Tomography/Computed Tomography Imaging

**DOI:** 10.4274/mirt.24008

**Published:** 2016-02-10

**Authors:** Senait Aknaw Debebe, Mohammed Goryawala, Malek Adjouadi, Anthony J. Mcgoron, Seza A. Güleç

**Affiliations:** 1 Florida International University, College of Engineering and Computing, Department of Biomedical Engineering, Miami, USA; 2 Florida International University, College of Engineering and Computing, Center for Advanced Technology and Education, Miami, USA; 3 Florida International University, Herbert Wertheim College of Medicine, Department of Surgical Oncology, Miami, USA

**Keywords:** 18F-fluorothymidine, 18F-fluorodeoxyglucose, positron emission tomography/computed tomography, pancreatic cancer imaging, image processing, tumor proliferative activity, tumor glycolytic activity

## Abstract

**Objective:**

This phase-I imaging study examined the imaging characteristic of 3’-deoxy-3’-(^18^F)-fluorothymidine (^18^F-FLT) positron emission tomography (PET) in patients with pancreatic cancer and comparisons were made with (^18^F)-fluorodeoxyglucose (^18^F-FDG). The ultimate aim was to develop a molecular imaging tool that could better define the biologic characteristics of pancreas cancer, and to identify the patients who could potentially benefit from surgical resection who were deemed inoperable by conventional means of staging.

**Methods:**

Six patients with newly diagnosed pancreatic cancer underwent a combined FLT and FDG computed tomography (CT) PET/CT imaging protocol. The FLT PET/CT scan was performed within 1 week of FDG PET/CT imaging. Tumor uptake of a tracer was determined and compared using various techniques; statistical thresholding (z score=2.5), and fixed standardized uptake value (SUV) thresholds of 1.4 and 2.5, and applying a threshold of 40% of maximum SUV (SUV_max_) and mean SUV (SUV_mean_). The correlation of functional tumor volumes (FTV) between ^18^F-FDG and ^18^F-FLT was assessed using linear regression analysis.

**Results:**

It was found that there is a correlation in FTV due to metabolic and proliferation activity when using a threshold of SUV 2.5 for FDG and 1.4 for FLT (r=0.698, p=ns), but a better correlation was obtained when using SUV of 2.5 for both tracers (r=0.698, p=ns). The z score thresholding (z=2.5) method showed lower correlation between the FTVs (r=0.698, p=ns) of FDG and FLT PET.

**Conclusion:**

Different tumor segmentation techniques yielded varying degrees of correlation in FTV between FLT and FDG-PET images. FLT imaging may have a different meaning in determining tumor biology and prognosis.

## Abstract (tr)

**Amaç:**

Bu faz I çalışma 3’-deoxy-3’-(^18^F)-fluorothymidine (18F-FLT) pozitron emisyon tomografisi (PET) görüntülemesinin pankreas kanseri hastalarındaki karakteristiklerini araştırmış ve sonuçlarını (^18^F)-fluorodeoxyglucose (^18^F-FDG) görüntülemesi ile karşılaştırmıştır. Bu çalışmadaki nihai amaç, pankreas kanserinin biyolojik özelliklerini tanımlayan ve pankreas kanseri hastaları arasında standart anatomik görüntüleme yöntemleri ile cerrahi tedavi imkanı olmadığı sonucuna varılan hastalarda cerrahi endikasyonlarının ve sınırlarının yeniden tanımlanmasını sağlayacak bir moleküler görüntüleme yöntemi geliştirmektir.

**Yöntem:**

Yeni teşhis almış ve hiçbir tedavi görmemiş altı hasta bir hafta ara ile FLT ve FDG bilgisayarlı tomografi (BT) PET/BT çalışmasına alındı. Tümör radyofarmasötik tutulumu tespit edilerek değişik yöntemlerle karsılaştırıldı. İstatistiki eşikleme (z skoru=2,5), sabit standart tutulum değeri standardized uptake value (SUV) olarak 1,4 ve 2,5, ve maksimum SUV değerinin %40’ı düzeyinde eşikleme yöntemleri denendi. FLT ve FDG ile fonksiyonel tümör hacmi hesaplamaları yapıldı ve ilişkileri lineer regresyon analizi ile araştırıldı.

**Bulgular:**

FDG için 2,5 SUV ve FLT için 1,4 SUV değerleri kullanıldığında, FDG ve FLT fonksiyonel tümör hacimleri arasında bir korelasyon bulundu (r=0.698, p=ns). Her iki radyofarmasötik için 2,5 SUV değeri eşik alındığında ise fonksiyonel tümör hacimleri arasında daha iyi bir korelasyon saptandı (r=0.698, p=ns). Z skoru eşikleme yöntemiyle FLT ve FDG fonksiyonel tümör hacimleri arasında zayıf bir ilişki tespit edildi (r=0,698, p=ns).

**Sonuç:**

Değişik tümör segmentasyon yöntemleri FLT ve FDG fonksiyonel tümör hacimleri arasında değişik derecelerde korelasyon gösterdi. FLT görüntülemesi tümör biyolojisinin belirlenmesinde farklı bir anlamı ve prognostik değeri bulunabilir.

## INTRODUCTION

Pancreatic cancer (ductal adenocarcinoma) accounts for approximately 36,800 deaths per year in United States (1). The majority of patients present in the late stages of the disease with locally advanced or metastatic tumors, among which only 10 to 20% of patients are candidates for resection and hence have any potential for cure. The signs and symptoms of pancreatic cancer vary from vague, nonspecific abdominal complaints to severe jaundice and the diagnosis can often be difficult, especially in the early stages (2). Despite improvements in diagnostic technology and development of new systemic therapy agents, the prognosis of the disease has not shown much improvement. Surgical resection is the only potential curative treatment available for patients with pancreatic cancer (1).

(^18^F)-fluorodeoxyglucose (FDG) positron emission tomography (PET)/computed tomography (CT) PET/CT has now become a standard imaging technique for most cancers and the majority of cancers exhibit increased glucose metabolism resulting in high concentration of ^18^F-FDG in lesions. FDG-PET can change the management of pancreatic cancer by revealing unsuspected metastases to liver, bone and lungs, thereby avoiding the morbidity and mortality of unnecessary surgical interventions (3). Proliferative activities of tumors are known to correlate with prognosis (4). Numerous markers have been described to predict the biological behavior of tumors and outcomes following surgical and medical treatment. Ki-67 is a nuclear antigen present only in the nuclei of proliferating cells and Ki-67 immunohistochemistry has been used to evaluate tumors’ proliferative activity (4,5,6). Clinical evaluation and quantification of proliferative activity and tumor invasiveness can be performed using 3’-deoxy-3’-(^18^F)-fluorothymidine (^18^F-FLT) PET imaging (7,8,9,10,11). 18F-FLT works as a terminator of the growing DNA chain (12). Actually little ^18^F-FLT is accumulated in DNA, it is rather retained intracellularly after phosphorylation by thymidine kinase 1. This is very much analogous to the imaging of the glucose pathway with ^18^F-FDG after trapping by hexokinase. Both compounds therefore reflect accumulation by transport and subsequent activation by the first step in the utilization pathways. However, ^18^F-FLT does not reflect the whole of DNA synthesis just as ^18^F-FDG does not reflect the whole of glucose use.

In this study, we examined the imaging characteristics of pancreatic cancer patients to determine the correlation between the metabolic and proliferative activity of pancreatic cancer using FDG and FLT PET images, respectively. The parameters of interest were functional tumor volume (FTV), Total glycolytic index (TGI) and Total proliferative index (TPI). FTV, TGI and TPI were determined from both FDG and FLT PET images. These parameters measure the metabolic and proliferation activity of tumors using FDG and FLT PET/CT images, respectively, which have clinical value in the assessment of tumor biology, prognosis, response to treatment evaluation, and patient selection for therapeutic interventions (4).

## MATERIALS AND METHODS

### Inclusion Criteria

Patients with pancreatic and periampullary tumors were identified by pathological examination. Those who were potential candidates for the trial were further assessed for eligibility. Inclusion criteria were: clinically diagnosed pancreatic cancer (newly diagnosed as well as those under treatment), age ≥18, ability and willingness to give a written consent, life expectancy >3 months and Karnofsky performance status ≥70. Patients with age <18, inability or unwillingness to give a written consent, life expectancy <3 months, Karnofsky performance status <70, pregnant or nursing women (urine pregnancy test was performed prior to the investigational radiotracer injection) and individuals allergic to FLT were excluded.

### Patient Characteristics

Six patients fulfilling the inclusion criteria were enrolled in the study of which two were females and four were males (median age of 61.5 and range 56-71 years). The demographics of the patients with the estimated anatomic pancreatic volumes are presented in Table 1. The anatomic pancreatic volumes are estimated by outlining the pancreas on abdominal CT images by an expert radiologist.

### Positron Emission Tomography/Computed Tomography Imaging

The study was performed under a Food and Drug Administration approved Investigational New Drug and after institutional review board review and approval. The 3’-^18^F-fluoro-3’-deoxy-L-thymidine used in this trial was obtained from Cardinal Health 414, LLC. Administered activities both for ^18^F-FLT and ^18^F-FDG were 10±1 mCi with post injection imaging point of 60±15 minutes. Images were obtained with 16-slice Siemens PET/CT camera. The scanning parameters for the CT imaging were 140 kVp, 80mA, 0.5s rotation time and 512×512-pixel matrix. CT image sizes range from 512x512x186 to 512x512x273. PET image sizes range from 168x168x186 to 168x168x273 with voxel size of 4.0627x4.0627x4 mm3.

### Methods for Finding Functional Tumor Volumes

As a first step, FLT PET images were manually co-registered with FDG PET/CT images using AMIDE software, a free tool for viewing, analyzing and registering volumetric medical imaging data sets. Next, an experienced board certified radiologist used the region of interest (ROI) method to isolate the pancreas from CT images of the FDG PET/CT. After this point, MATLAB^®^ (The MathWorks Inc.) was used to perform automatic tumor segmentation. The Binary masks from the ROIs of CT images were mapped to the co-registered FDG PET and FLT PET images to segment the pancreas. Third party interactive application software called ScanIPTM was used in 3D medical image analysis to estimate the volume of the segmented pancreas. Once the pancreas was segmented from the two images, different thresholding techniques were applied to find the FTVs. We have used statistical and SUV threshold techniques.

 Our aim in this study was not to validate different tumor delineation methods, but rather to determine the FTV, TGI and TPI relationships due to the uptake of ^18^F-FDG and ^18^F-FLT by using well practiced fixed threshold methods.

### Statistical Tumor Segmentation

The segmented pancreas was normalized by z score transformation using equation 1, where ‘x’ is the pixel intensity value, and ‘μ’ and ‘σ’ are the mean and standard deviation of the segmented pancreas images, respectively. The z score transformation procedure for normalizing data is a familiar statistical method in neuroimaging and psychological studies (3). This method converts the original pixel intensity image to a probability map that represents deviations from the normal using voxel-by-voxel comparison, which facilitates image interpretation.

The z score, which transforms the image into a statistical parametric map, was calculated for each pixel. Threshold of z≥2.5 was then applied which only highlights those pixels which can confidently be labelled as active, i.e. those areas which deviate significantly from the normal (13). Using this technique considers the low tumor to background ratio by comparing each pixel to the surrounding pixels through deviation from the mean.

### Standardized Uptake Value (SUV) Based Tumor Segmentation

SUV provides biological and functional activity of a tumor (14). Quantification of FLT with SUV provides information about cells undergoing active proliferation while SUV of FDG provides information on increased glucose metabolism. SUVs were calculated with mathematical expression shown in equation 2.

Different SUV thresholds were used to investigate tumor localization; SUV of 1.4, 2.5, 40% SUV_max_ and SUV_mean_. SUV_max_ and SUV_mean_ refer to the maximum and mean SUV values from each individual patient’s PET images. One of the methods was using 40% of the maximum SUV uptake for each patient to segment tumor. Each patient has different maximum SUV uptakes for the radiotracer type, thus the amount of threshold varies accordingly. From the maximum SUVs, 40% of this value (40% SUV_max_) was applied as a threshold to segment tumors. The same concept was used when the mean SUV (SUV_mean_) uptake for tumor segmentation was applied. Table 1 shows the maximum SUVs for each patient.

### Functional Tumor Volume Measurement

The voxels, which exceed the applied threshold value, were counted to find the FTV using equation 3 where AV is the active voxel that remained after applying the threshold. Volume of a voxel was 4.0627x4.0627x4 mm^3^. The FTVs for ^18^F-FLT and ^18^F-FDG were then assessed using linear regression analysis.

### Total Glycolytic and Total Proliferative Index Measurements

The TGI/TPI is the product of functional volume and tumor SUV_max_. The FTV were multiplied with the respective SUV_max_ of the patient.

## RESULTS

**Figure 1:**
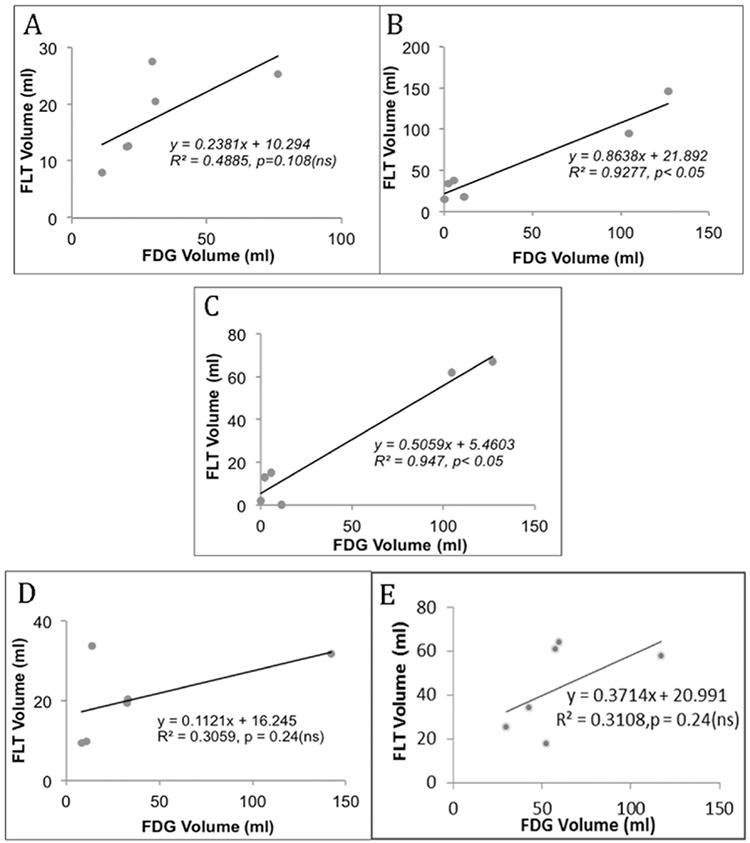
Functional tumor volume relationships using (A) z score thresholding, (B) SUV cutoffs of 2.5 for FDG and 1.4 for FLT (C) SUV cutoffs of 2.5 for both FDG&FLT (D) 40% of SUV_max_ thresholding (E) SUV_mean_ thresholdFDG:Fluorodeoxyglucose, FLT: Fluorothymidine

**Figure 2:**
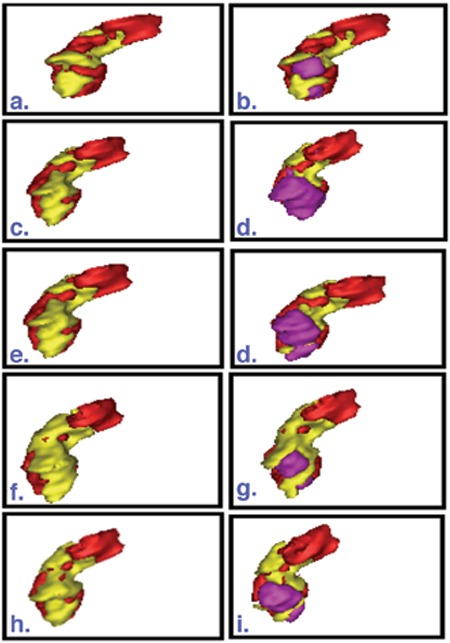
3D rendered volume of pancreas (red) and tumor volume delineated based on the uptake of only FDG (yellow) (left), and FDG and FLT (pink) superimposed (right) for patient 3 using (a)-(b) z score, (c)-(d) SUV of 2.5 (FDG) & 1.4 (FLT), (e)-(f) SUV of 2.5 for both tracers, (f)-(g) 40% SUV_max_ and (h)-(i) SUV_mean_ thresholding

Results of FTVs (ml) using the different tumor segmentation methods on ^18^F-FDG and ^18^F-FLT PET images are presented in Table 2 (A) and (B) respectively. Linear regressions were performed between estimated FTV from ^18^F-FDG and ^18^F-FLT PET for the five different thresholding techniques shown in Figure 1. Correlation coefficients (r), t and p values of the linear regression were reported. Analyses were performed two-sided at a 5% level of significance. Figure 2 shows the 3D rendered images of the pancreas and segmented tumors using the five different threshold techniques for patient 3.

FDG and FLT PET images with average maximum SUV uptake of 9.1 (median 5.38, range 1.32-33.21) and 8.1 (median 7.15, range 2.6-16.52), respectively, were analyzed. Results showed a correlation in FTV due to metabolic and proliferation activity. Using a threshold of SUV 2.5 for FDG and 1.4 for FLT, a correlation coefficient (r) of 0.9606 (t value=7.16, p value <0.05) was found. A slightly better correlation was found with SUV of 2.5 for both tracers with of r=0.973 (t value=8.46, p value <0.05). The p values (5% significance level) from the two SUV methods strongly support that there is a correlation in FTV from ^18^F-FDG and ^18^F-FLT PET scans.

Strong correlation between TPI and TGI was found using 40% of SUV_max_, r of 0.9977 (t value=29.37, p<0.05). The 2nd best correlation between TPI and TGI was observed when threshold of SUV 1.4 for FLT and 2.5 for FDG used with r=0.9427(t value=5.65, p value <0.05).

The z score threshold (z=2.5) method showed a moderate correlation between FTVs (r=0.698, t value=1.95, p value=ns), and TGI and TPI (r=0.89, t value=3.9, p value <0.05) between the two images. The p values support the null hypothesis that there is no correlation between the FTVs of FDG and FLT PET images with z score method.

## DISCUSSION

There is no universally validated technique for tumor delineation, and manual segmentation is biased by the experience of the nuclear physician and the contouring protocol used. Thureau et al. (15) has suggested using a fixed threshold of SUV 1.4 for ^18^F-FLT uptake due to the low tumor-to-background ratio in PET images of lung cancer. In addition, it was also demonstrated that using SUV of 1.4 gives a similar result as the method used by physicians to delineate tumors. It is also explained in the literature that the difference in tumor volumes using different SUV thresholds to segment ^18^F-FDG uptake was insignificant. Han et al. (16) also concluded that SUV cutoff of 1.4 for ^18^F-FLT PET/CT and SUV of 2.5 for ^18^F-FDG PET/CT provided the best estimate of gross tumor volume. Hellwig et al. (17) demonstrated that SUV of 2.5 thresholds for 18F-FDG gives a high overall accuracy for clinical images. In our study, SUV cutoff values that showed to be reproducible were used for tumor segmentation. The resulting volumes from ^18^F-FDG and ^18^F-FLT PET images were compared for correlation.

In our study, the FLT PET images demonstrated physiologic uptake in the liver and bone marrow. The activity in the normal pancreatic tissue was at the background level. FLT uptake in the tumors was at variable intensity and did not encompass the entirety of the FDG-positive regions in the tumor’s topography. Determining FTV from FLT PET images is challenged by partial volume effect (in small tumors), and is subject to errors from manual tumor segmentation that might result in underestimation. These drawbacks have been explained as a reason for negative results in FLT PET scans (18). The FTV from FDG PET images could also be potentially challenged by the enhancement of FDG activity from a peritumoral inflammatory response. Thus, a technique that is ideal for FDG images might not necessarily be applicable to FLT images. To address these technical difficulties, we used semiautomatic segmentation methods. The primary aim of this study was to assess and compare the topography and size of FTV from proliferation (FLT PET) and metabolic (FDG PET) images.

In this study, the pancreas was first segmented from ^18^F-FDG CT scans by an experienced radiologist. The segmented pancreas was then mapped to co-registered ^18^F-FDG and ^18^F-FLT PET images. Different thresholding techniques were applied to automatically segment tumors from the PET scans.

We used two major methods; statistical and SUV methods. To our knowledge, this study is the first to use a statistical (z score) and fixed SUV thresholds on FLT and FDG PET/CT images of pancreatic cancer in tumor segmentation. The use of z score threshold considers all pixels inside the pancreas and delineates only those pixels that deviate considerably from the mean activity inside the pancreas. In the case of FLT PET images that have very low contrast, this method provides an excellent way to differentiate between background and tumor pixel intensities. Z scores on FLT images take into consideration the overall organ uptake since not every organ has similar cell proliferation rates. This method might not provide an alternate choice for FDG PET images where tumor to background uptake is well segregated.

The SUV cutoffs (2.5, 1.4, 40% SUV_max_ and SUV_mean_) presented here to segment tumor volumes have been tested to be reproducible in tumor volume delineation (15,17,19). We have found different FTVs from FDG and FLT PET images, which might result in different treatment planning and different dose delivery if either of the two tracers is used for diagnostics workup. This might imply that giving a treatment solely based on FLT or FDG uptake might be misleading suggesting that incorporating the two images can be beneficial in treatment planning.

There is a satisfactory FTV correspondence between FDG and FLT PET when SUV cutoffs of 2.5 and 1.4, respectively, are used for tumor segmentation. The correlation between TGI and TPI is seen to be high when a threshold of 40% of SUV_max_ is used but this method also gave the highest probability of false prediction.

Though the volumes delineated as a tumor from the two images do not always occur in the same place of the pancreas, our study showed that FTVs from ^18^F-FDG and ^18^F-FLT PET images do have correlation. In most patients, it was observed that tumors segmented from FLT PET images occur at the head of the pancreas (Figure 2). Thureau et al. (15) postulated that proliferative volume should not be greater than metabolic volume. In our study, the z score method yielded higher tumor volumes from ^18^F-FDG than ^18^F-FLT PET images in all patients unlike the SUV methods where this result was variable from patient to patient.

In conclusion, the FTVs correlation seen between FDG and FLT PET scans depends on the type of tumor segmentation technique used. SUV based thresholds showed correlations in the FTVs but z score method showed no correlations of FTVs between FDG and FLT PET scans.

## CONCLUSION

Different tumor segmentation techniques yielded varying degree of correlation in functional tumor values between FLT and FDG PET images. The statistical threshold technique showed higher tumor volumes from FDG images than from FLT PET images in all patients. Due to the limited number of patients and the lack of a gold standard, further investigation is required to fully appreciate correlations in tumor topography and size between ^18^F-FDG PET and ^18^F-FLT PET images.

## Figures and Tables

**Table 1:**
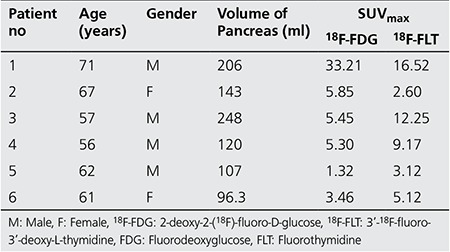
Patient characteristics

**Table 2A:**
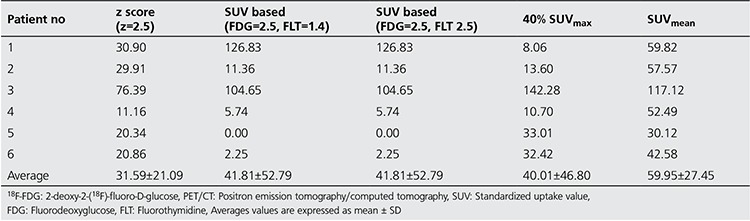
Estimated pancreas tumor volumes using (A) 18F-FDG PET/CT Imaging

**Table 2B:**
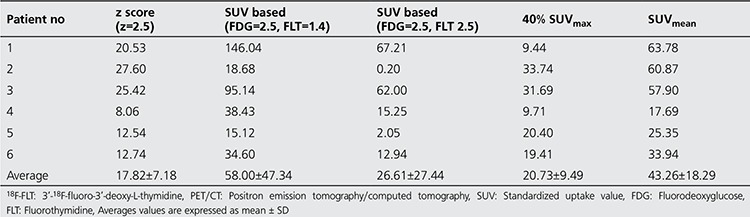
Estimated pancreas tumor volumes using (B) 18F-FLT PET/CT Imaging
